# Antihyperglycemic mechanism of metformin occurs via the AMPK/LXRα/POMC pathway

**DOI:** 10.1038/srep08145

**Published:** 2015-01-30

**Authors:** Kumsun Cho, Jae Yong Chung, Sung Kweon Cho, Hyun-Woo Shin, In-Jin Jang, Jong-Wan Park, Kyung-Sang Yu, Joo-Youn Cho

**Affiliations:** 1 Department of Clinical Pharmacology and Therapeutics, Seoul National University College of Medicine and Hospital, Seoul, Republic of Korea; 2 Department of Biomedical Science, Seoul National University College of Medicine, Seoul, Republic of Korea; 3 Department of Clinical Pharmacology and Therapeutics, Seoul National University College of Medicine and Bundang Hospital, Seongnam, Republic of Korea; 4 Department of Pharmacology, Yonsei University College of Medicine, Seoul, Republic of Korea; 5 Department of Pharmacology, Ischemic/Hypoxic Disease Institute, Seoul National University College of Medicine, Seoul, Republic of Korea; 6 Cancer Research Institute, Seoul National University College of Medicine, Seoul, Republic of Korea

## Abstract

Metformin is a first-line drug for treating type 2 diabetes. Although metformin is known to phosphorylate AMP-activated protein kinase (AMPK), it is unclear how the glucose-lowering effect of metformin is related to AMPK activation. The aim of this study was to identify the urinary endogenous metabolites affected by metformin and to identify the novel underlying molecular mechanisms related to its anti-diabetic effect. Fourteen healthy male subjects were orally administered metformin (1000 mg) once. First morning urine samples were taken before and after administration to obtain metabolomic data. We then further investigated the anti-diabetic mechanism of metformin *in vitro* and *in vivo*. The fluctuation of the metabolite cortisol indicated that the neuroendocrine system was involved in the anti-diabetic effect of metformin. Actually we found that metformin induced AMPK/liver X receptor α (LXRα) phosphorylation, followed by pro-opiomelanocortin (POMC) suppression in rat pituitary cells. We confirmed this result by administering metformin in an animal study. Given that cortisol stimulates gluconeogenesis, we propose the anti-hyperglycemic effect of metformin is attributed to reduced POMC/adrenocorticotropic hormone (ACTH)/cortisol levels following AMPK/LXRα phosphorylation in the pituitaries.

Metformin is frequently prescribed for type 2 diabetes and has many of advantages including it is weight neutral^1^, and it does not affect the risk of cardiovascular disease^2^ and hypoglycemia^3^. Metformin exerts its antihyperglycemic action primarily by inhibiting hepatic gluconeogenesis and by increasing the action of insulin in certain target organs, like muscle^4^ and fat^5^. Additionally, metformin is progressively used in polycystic ovary syndrome (PCOS) and many studies suggest that metformin could affect pituitary gonadotropin-secreting cells^6^. However, the underlying mechanism by which metformin regulates blood glucose levels and/or affects pituitary remains unknown.

Recently, metabolomics has progressed remarkably within the past decade and provided mechanistic insights by correlating biochemical changes with phenotypes. An untargeted metabolomics approach is especially comprehensive in scope and can measure as many metabolites as possible from biological samples simultaneously, without bias. This has great potential for revealing the underlying mechanism of pathophysiology or drug effects. Therefore, these ‘metabolomic' studies are seen as a useful tool for the study of metabolic diseases (e.g., diabetes and polycystic ovary syndrome (PCOS)) to investigate systemic alterations in metabolism (e.g., high blood sugar and hormone imbalance) or mechanism of its therapeutic drugs (e.g., metformin).

It is well known that the pleiotropic actions of metformin are associated with AMP-activated protein kinase (AMPK)^7^. The pituitary-mediated actions of metformin were also elucidated in PCOS and diabetes. In particular, Lucie Tosca et al. elucidated that metformin-induced AMPK activation could exert its action in pituitary cells^6^. It was also supported by recent studies that orally-dosed metformin rapidly crosses the blood-brain-barrier (BBB) and accumulates in the pituitary gland and hypothalamus of rats^8^. However, the anti-hyperglycemic mechanism of metformin, associated with the neuroendocrine system, is not fully understood.

In this study, we used untargeted metabolomics and identified the changes in urinary endogenous metabolites after metformin administration in human subjects. Subsequently, we uncovered the novel anti-hyperglycemic mechanism of metformin involving AMPK activation through *in vitro* and *in vivo* studies.

## Results

**Figure 1:**
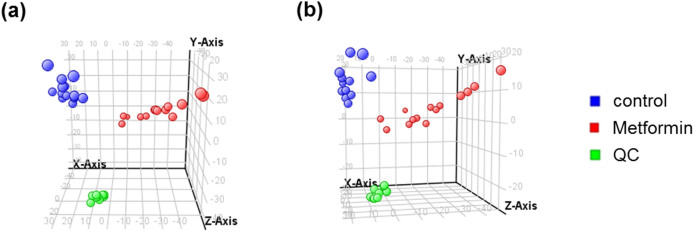
Untargeted metabolomic profiling using HPLC/Q-TOF MS generated PCA score plots discriminating the metformin treated healthy subjects group (*red*) from the control group (*blue*). Cs are for monitoring the overall quality of the analysis procedure (*green*). Data are shown for (a) the positive and (b) the negative ESI datasets. A representative data from 3 independent experiments.

In this study, 14 healthy subjects were administered metformin once orally, and first morning urine samples were obtained before and after treatment. The samples were analyzed by HPLC/Q-TOF MS and further by multivariate data analysis. Principal component analysis (PCA) revealed a clear discrimination between the control (*blue*) and metformin-treated group (*red*) in both ESI (+) and (−) modes, which indicates that metformin could affect the endogenous metabolomic profiles in urine (Figure 1).

**Figure 2:**
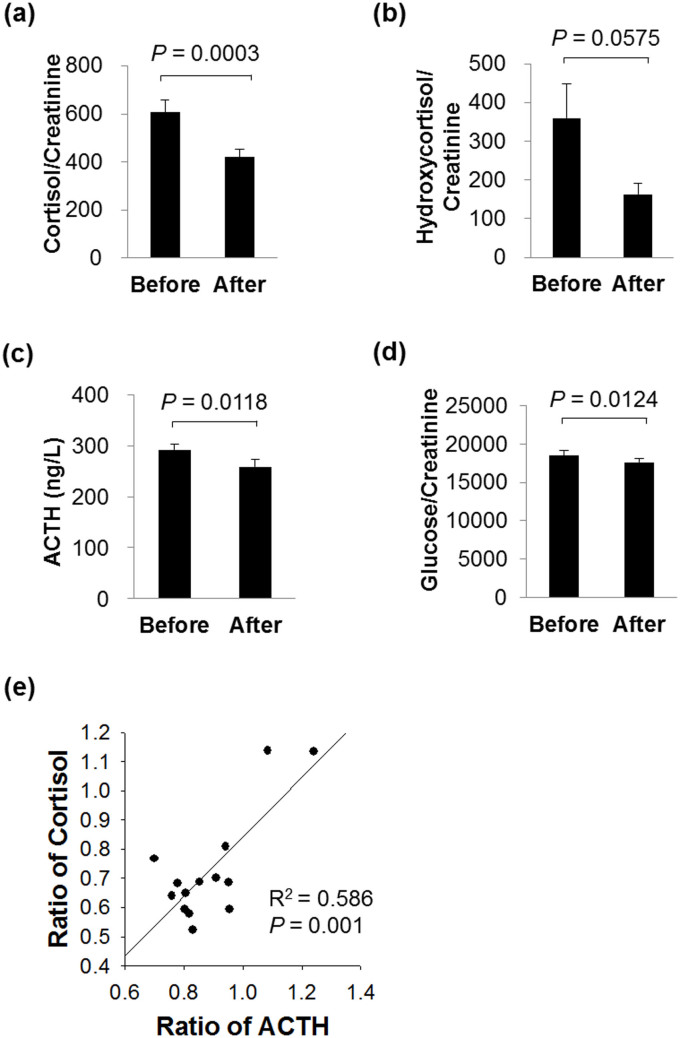
Metformin reduces urinary cortisol, hydroxyl cortisol, ACTH, and glucose levels in the subjects. For quantification, (a) cortisol and its metabolite (b) hydroxycortisol, (c) ACTH, and (d) glucose levels were normalized to those of creatinine. Levels of ACTH were measured by ELISA. Data are expressed as the mean ± SE. (e) Correlation between the ratio of cortisol and ACTH levels showed that metformin reduced ACTH secretion and cortisol levels. A representative data from 3 independent experiments.

The urine levels of several endogenous metabolites were changed significantly by orally administrated metformin (see Supplementary Table S1 online). Especially, four metabolites including cortisol, retinyl β-glucuronide, betaine, and cholic acid glucuroninde were identified with pure analytical standards, and finally quantified by normalization for urinary creatinine (see Supplementary Fig. S2 online). Among them, cortisol was significantly decreased and its metabolite hydroxycortisol also decreased after metformin administration, although this difference was a borderline significant trend (Figure 2a and 2b). Also most subjects' levels of cortisol were decreased (see Supplementary Fig. S3a online). Cortisol is a well-known stress hormone, and plays key roles in stimulating gluconeogenesis by breaking down of glycogen to glucose-1-phosphate and glucose^9^. Given that cortisol has been known that which is closely related to the glucose levels, we further studied how cortisol effects on antiglycemic action of metformin.

Since the single treatment of metformin rapidly affects cortisol levels, we then observed the neuroendocrine response to metformin. Previous studies have examined enhanced HPA axis activity in patients with type 2 diabetes^10^,^11^. However, given that metformin is prescribed primarily as an antidiabetic drug, its role in the HPA axis and related mechanisms is not clear. Thus, we hypothesized that reduced cortisol was related to the antidiabetic function of metformin. At first, considering that ACTH regulates secretion of cortisol by stimulating the adrenal cortex, we measured urinary ACTH concentrations in human subjects groups by using ELISA analysis. The urinary ACTH concentrations were decreased by 11% in metformin administered group (Figure 2c). In addition, the changes in cortisol and ACTH levels correlated significantly with each other (Figure 2e). Furthermore, glucose levels were also decreased (Figure 2d). These data showed that metformin could reduce ACTH and cortisol secretion as well as glucose level. To further investigate how metformin reduces cortisol through the neuroendocrine system, we evaluated key elements of the HPA axis *in vitro* and *in vivo* after metformin treatment.

Matsumoto et al recently reported that pituitary LXRα plays an important role in cortisol levels through the regulation of POMC and ACTH by the LXR agonist in mice. Particularly, they showed that LXRα directly regulates the POMC gene promoter, indicating that POMC gene expression is positively regulated by LXRα at the transcriptional level^12^. In addition, Hwahng et al reported that AMPK phosphorylated LXRα at threonine residues has an inhibitory effect on those downstream genes^13^.

**Figure 3:**
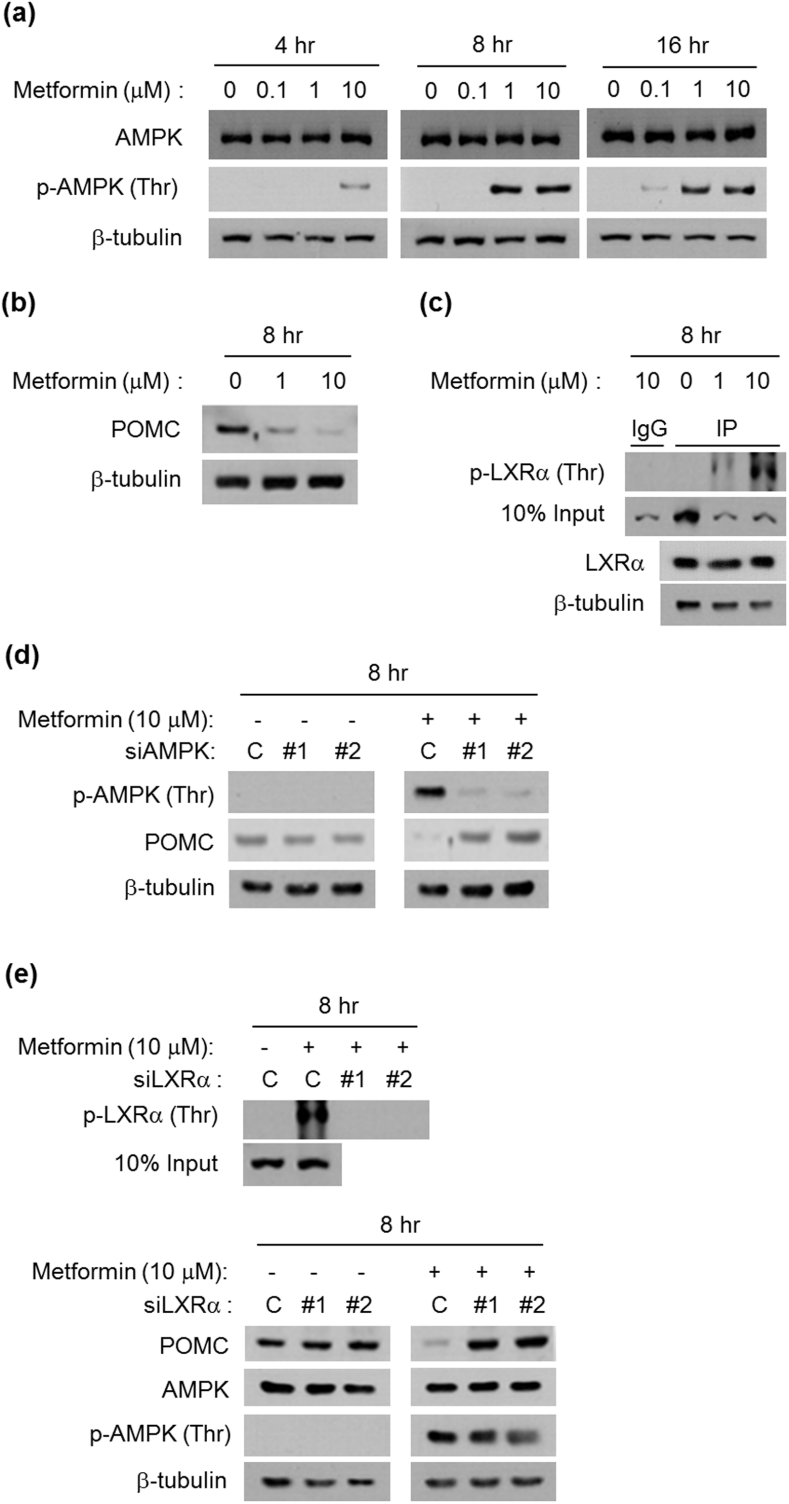
Metformin suppresses POMC protein levels through phosphorylation of AMPK and subsequently LXRα *in vitro*. The rat pituitary adenoma GH3 cells were treated with metformin by using the indicated concentrations and treatment times, and total cell lysates were used for western blotting. (a and b) Metformin upregulated AMPK phosphorylation and downregulated POMC expression. (c) Total cell lysates were used for immunoprecipitation with anti-phopho-Thr antibody and western blotting with anti-LXRα antibody. (d) Reduced POMC after metformin treatment was restored when two siRNAs (#1 and #2) targeting AMPK were transfected in GH3 cells. (e) After metformin treatment, knockdown of LXRα by using two siRNAs (#1 and #2) targeting LXRα (*upper*) restored POMC expression, although p-AMPK was still enhanced (*lower*). A representative data from 3 independent experiments. Full-length blots are presented in Supplementary Figure S8.

A recent report on metformin quantification in the rat brain found that orally-dosed metformin rapidly crossed the blood-brain-barrier (BBB) and affected POMC (a precursor of ACTH) expression in the pituitary as well as ACTH-stimulated cortisol levels in blood^8^. In addition, other researchers stated that metformin activates AMPK in rat pituitary cells^6^. Here, we investigated, in rat pituitary adenoma cells, that metformin induces AMPK activation by phosphorylating in early time (Figure 3a and Supplementary Fig. S4). We also found reduced POMC expression at the dose and time when AMPK was fully activated (Figure 3b). Then we measured threonine phosphorylated LXRα and found that the inhibitory phosphorylation is upregulated in the presence of metformin, although total LXRα expression was not changed (Figure 3c). Using knockdown techniques, we found out that metformin-induced POMC reduction is dependent on AMPK and LXRα activation; however, metformin induced AMPK activation occurs independently from LXRα phosphorylation (Figure 3d and 3e). This result indicated that metformin induced AMPK activation reduces POMC expression through LXRα inhibitory phosphorylation in the pituitary.

**Figure 4:**
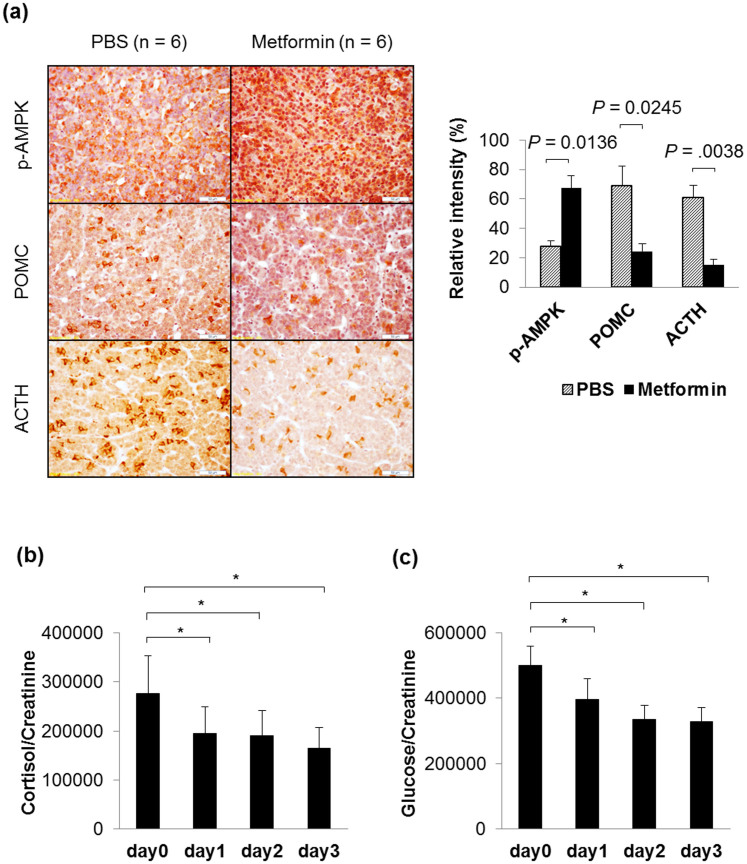
Metformin activates AMPK and reduces POMC, ACTH, cortisol, and glucose levels *in vivo*. (a) Immunohistochemical staining (*dark brown*) of paraffin-embedded pituitary sections showed metformin (20 mg/kg) induced AMPK phosphorylation, and inhibited POMC and ACTH expression (*left*). The images are of a representative section (original magnification, ×400. Bar, 50 μm). The number of cells immunoreactive for p-AMPK, POMC, or ACTH was normalized to the total number of cells (*right*). Data represent the mean ± SE (n = 6). Relative quantification of the creatinine normalized urinary (b) cortisol and (c) glucose in rats before and after metformin treatment (once-daily for 3 consecutive days). Data are expressed as the mean ± SE (n = 6). *P < 0.05, the Wilcoxon signed ranks test used.

**Figure 5:**
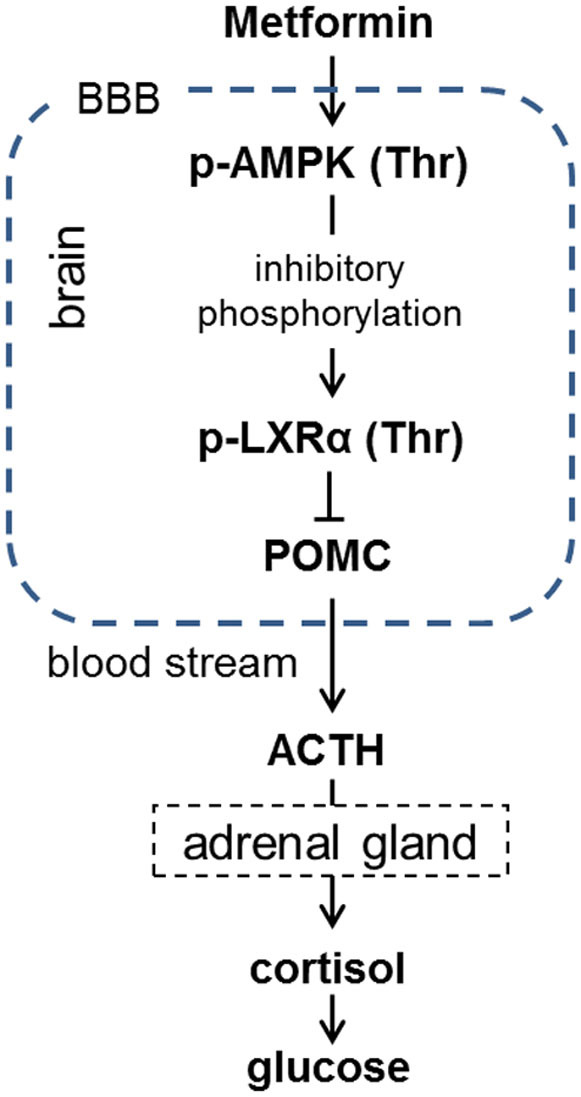
Proposed mechanism of the antihyperglycemic action of metformin action via the AMPK/LXRα/POMC pathway.

To evaluate the *in vivo* effects of metformin on cortisol reduction through the AMPK/LXRα/POMC pathway, we administered metformin to rats in this study. We verified that p-AMPK, POMC, and ACTH expression levels were significantly altered in the pituitary glands of rats (Figure 4a), and the urinary cortisol levels also decreased after administration of metformin (Figure 4b). The protein expression of p-AMPK increased, while POMC and ACTH expression decreased in the metformin-administered group. We also confirmed, with immunostaining, LXRα and LXRβ in the pituitary by using antibodies specific to each of them (see Supplementary Fig. S5 online). Using immunohistological analysis of the rat pituitary, we elucidated that the number of LXRα immunoreactive cells was greater than that of LXRβ reactive cells, and that the role of LXRα is critical in the pituitary. These results are supported by a study that found LXRα is more highly expressed than LXRβ in the pituitary and directly regulates POMC at the transcriptional level^12^. We also found the rat urinary cortisol and glucose levels were not changed in the PBS treatment group (data not shown); however, they changed significantly in the metformin treatment group, similar to in humans (Figure 4b and 4c). Therefore, metformin induced ACTH reduction via the AMPK/LXRα/POMC pathway, and simultaneously reduced cortisol and glucose *in vivo* (Figure 5).

## Discussion

We found that several urine metabolites were significantly changed in metformin administered healthy subjects using metabolomic analysis. This urinary metabolomics study determined the endogenous metabolites affected by metformin administration. Among them, retinyl β-glucuronide and cholic acid glucuronide are conjugates of metabolites of retinol and bile acid, respectively. Given that retinol and bile acid induce liver toxicity^14^,^15^, we considered that increased glucuronidation of them provides clues about the potential detoxifying role of metformin. Furthermore, recent studies stated that metformin could treat chronic liver diseases by protecting against bile acid induced apoptosis^16^. Another metabolite found, betaine, is known to lower homocysteine levels in blood as an antioxidant^17^. Given that metformin treatment elevates homocysteine levels, even under short-term treatment conditions, we inferred that reduced levels of betaine have causative effects on metformin induced oxidative stress^18^,^19^. However, the clinical significance of these findings remains to be further investigated. Finally, we identified cortisol, a well-known stress hormone that is secreted from the adrenal cortex and stimulated by adrenocorticotropic hormone (ACTH) through a major part of the neuroendocrine system hypothalamic-pituitary-adrenal (HPA) axis. Surprisingly, our data showed that urinary cortisol, its metabolite hydroxycortisol, and ACTH levels were decreased after metformin administration, and all participants except two subjects showed the decreased cortisol and ACTH levels (see Supplementary Fig. S3a and S3b online).

Several studies reported that AMPK activation by either adiponectin or AICAR increased POMC expression or ACTH level. Iwasaki et al stated that AMPK activation by AICAR increased the activity of POMC gene^20^. However, they used different kinds of cells (AtT20 corticotroph cells) and conditions (starvation) contrary to our study. Actually, intracellular energy depletion with the resultant activation of AMPK directly stimulates the HPA axis at the pituitary level by increasing the expression of POMC gene. The activation of AMPK in high glucose (4.5 g/L) leaded to the suppression of POMC in our research (see Supplementary Fig. S6 online). Thus the glucose level or intracellular energy status could be a crucial switch of the AMPK-mediated POMC regulation. In other report, Guillod-Maximin et al addressed that adiponectin receptors were expressed in hypothalamus and colocalized with POMC neurons in rodents, and adiponectin could activate AMPK in the rat^21^. However, they did not show any direct relevance of POMC itself and the activation of AMPK by adiponectin. Furthermore, Qi et al showed that adiponectin suppressed glucose level significantly and reduced POMC expression in a dose-dependent manner although this is not statistically significant^22^. As above, this reduction of POMC under AMPK activation should be understood in terms of obesity or nutrient-sufficient status.

Given that LXRα regulates POMC gene expression in the pituitary^12^, we confirmed LXRα ligand increased POMC expression by using the synthetic LXRα ligand T0901317 and GW3965 hydrochloride. As shown in Supplementary Figure S6, both LXRα ligands upregulated the expression of POMC. However, when treated with metformin, POMC proteins were reduced although two kinds of LXR agonists were treated. Therefore, even in the presence of LXR agonist, metformin can be sufficient to inhibit LXRα via phosphorylating its threonine residue (see Supplementary Fig. S6 online). Taken together, it seems that the activation of AMPK by metformin could play a crucial role in POMC reduction under high glucose environment as like in the diabetic condition.

Hypothalamic AMPK has been suggested to act as a key sensing mechanism, responding to hormones and nutrients in the regulation of energy homeostasis. However, the precise neuronal populations and cellular mechanisms involved are unclear. AMPK plays a critical role as a sensor of cellular energy status in many organs including heart, adipose cell, liver, pancreatic beta cell, skeletal muscle, and brain^23^. When it comes to the role of hypothalamic AMPK in cellular energy regulation, it has long been studied that hypothalamic AMPK activation has been important as a regulator of energy homeostasis^24^. Appropriate counter-regulatory response is crucial for recovery from too low or high glucose level, and AMPK activation appears to mediate this function. In other words, AMPK acts differently according to its environmental glucose or other nutrients levels are high or low. Insulin-induced hypoglycaemia in rats increased AMPK phosphorylation and α2AMPK activity in the hypothalamus^25^. Recently, Claret et al found that hypothalamic AMPK plays a critical role in glucose sensing by using mice lacking AMPK in POMC and agouti-related protein-expressing neurons^26^. Also, they showed that the lack of AMPK in POMC neurons led mice to obesity because of their suppressed metabolic rate and increased feeding. However, the role of AMPK activation under hyperglycemic status has not been elucidated thoroughly. This point has a quite important meaning in terms of the role of AMPK activation by metformin in diabetic patients. Thus our finding that the activation of AMPK by metformin with plenty or enough of glucose would suppress POMC expression might provide a novel insight to the molecular mechanism of anti-diabetic action of metformin.

The reduced cortisol levels suggested that the rapid antihyperglycemic effect of metformin is attributed to the hypothalamic-pituitary-adrenal (HPA) axis, which we examined through *in vitro* and *in vivo* studies. We found the reduction of glucose, cortisol and ACTH resulted from the diminished POMC expression following AMPK and LXRα phosphorylation in the pituitary. In summary, the antidiabetic effect of metformin occurs via the AMPK/LXRα/POMC pathway. The AMPK activator metformin suppresses POMC and ACTH expression levels in rat pituitaries through inhibitory phosphorylation of LXRα (Figure 5).

To our knowledge, this is the first study to elucidate the antidiabetic mechanism of metformin, which decreases ACTH and cortisol by activating AMPK. Therefore, these findings increase our understanding of metformin which is the most widely used agent for the treatment and prevention of diabetes and insulin resistance syndrome by suggesting a novel antihyperglycemic mechanism of the drug. Furthermore, this study could alter existing mechanism of action of metformin, if confirmed and extended in further study. Also, this study provides the fundamentals for drug discovery and development of antidiabetic treatments targeting cortisol reduction.

## Methods

### Subjects and sampling

Fourteen healthy Korean male volunteers participated in the study (aged 20–50 years, weighing 50–90 kg, and having body mass indexes of 17–28 kg/m^2^). The study was in accordance with the Declaration of Helsinki and Korea Good Clinical Practice (KGCP) and the protocol and informed consent form were approved by the institutional review board (IRB) of Yonsei University Severance Hospital (4-2009-0334), Seoul, Korea, and each participant gave informed consent for the study. First morning urine samples were collected before and after oral administration of metformin (1000 mg at 8 PM) for metabolomic analyses (see Supplementary Fig. S1 online).

### Metabolomic profiling

A diluted (urine:water = 1:4) sample (5 μl) was loaded onto the column held at 40°C and eluted with 0.1% formic acid and 2 mM ammonium formate in water (solvent A), and 0.1% formic acid in methanol (solvent B) over 21 min. While maintaining a constant flow rate of 0.4 ml/min, the metabolites were eluted using the following gradient: 2–98% B from 0.1 to 13 min, and 98% B held constant for 2 min followed by a return to 2% B from 15.1 to 17 min. Chromatographic separations of metabolites in urine were performed with a Zorbax SB-C18, 50 × 2.1 mm, 1.8 μm (Agilent Technologies, Santa Clara, CA) analytical column using an Agilent 1200 series HPLC system (Agilent Technologies, Santa Clara, CA).

The eluent was introduced into Agilent 6530 quadrupole time-of-flight (Q-TOF) mass spectrometer (Agilent Technologies, Santa Clara, CA). The instrument settings were as follows: the nebulizer gas pressure and temperature were 30 psi and 325°C, respectively, and drying gas was set to 11 μl/min. The capillary voltage, capillary temperature, fragmentor voltage, and skimmer voltage were set to 3.5 kV, 300°C, 170 V, and 65 V, respectively. Centroid data were acquired over an *m/z* range of 100–1,100 using an accumulation time of 0.25 sec per spectrum. The mass accuracy and mass resolution were < 5 parts per million (ppm) and ~20,000, respectively. All raw data files were converted to the compound exchange file format using MassHunter DA reprocessor software (Agilent Technologies, Santa Clara, CA).

The overall quality of the analysis procedure was monitored using repeat extracts of a pooled urine sample (QC). To exclude metformin ions from the chromatographic mass data, the parent metformin ion was pursued both in ESI positive mode ([M + H]^+^ = 130.1087) and ESI negative mode ([M − H]^−^ = 128.0942) using MassHunter Qualitative Analysis software B.05.00 (Agilent Technologies, Santa Clara, CA), and it was also ensured that no metabolites or conjugates of metformin were present. The metformin ions listed above were removed and MassHunter Mass Profiler Professional software B.12.01 (Agilent Technologies, Santa Clara, CA) was used for aligning data and converting each metabolite feature (m/z × intensity × time) into a matrix of detected peaks versus compound identification. Each sample was normalized to the median of the baseline and log 2 transformed.

The resulting metabolites were identified using the human metabolome database (HMDB), METLIN, and the MS/MS fragment pattern with pure analytical standards. Finally, the metabolites were quantified by normalization to urinary creatinine concentrations.

### Cell lines and cell culture

The rat pituitary adenoma cell line GH3 was obtained from the Korean Cell Line Bank (Seoul, Korea). The pituitary adenoma cell line was authenticated by the suppliers by DNA profiling and cytogenetic analysis and in our laboratory by morphology and growth rate. Cells were grown under 5% CO_2_ at 37°C in Dulbecco's modified Eagle's medium supplemented with 10% fetal bovine serum (FBS), which was purchased from Sigma–Aldrich (St. Louis, MO, USA), along with other chemicals.

### Small interfering RNA (siRNA)-mediated gene silencing

For transient transfection with siRNAs, 40% confluent cells were transfected with negative control siRNA vector or two different siRNAs using Lipofectamine RNAiMAX reagents (Life Technologies, Grand Island, NY, USA). The transfected cells were allowed to stabilize for 36–48 h before being used in experiments.

### Animal experiments

All animal experiments were conducted in accordance with institutional guidelines and the protocol approved by the Seoul National University Institutional Animal Care and Use Committee (approval SNU-131002-2). Four-week-old male rats were randomly divided into the control or test group. Then, PBS or metformin (20 mg/kg; Sigma-Aldrich, St. Louis, MO) in PBS was administered intraperitoneally once-daily for 3 consecutive days to control or test groups, respectively. The dose (20 mg/kg) and treatment period (3 days) of metformin was determined considering the previous reports, which described that rodents need higher doses or longer period of metformin treatment than human due to the different drug sensitivity among species^7^,^27^,^28^,^29^. Rats were individually housed for 1 week prior to a 3-day acclimation period in the metabolic cages, and assessed for 3 days while fed a chow diet. Since rats are nocturnal animals that have the physiology during the day corresponds to the human physiology at night, we collected evening urine samples comparable to the morning sample to human^30^. The rats were euthanized with inhaled isoflurane and their pituitaries were removed and fixed in 4% paraformaldehyde overnight (see Supplementary Fig. S7 online).

Urinary cortisol levels were measured using a 12-chamber metabolic chamber system at the Institute for Experimental Animals (Seoul National University College of Medicine, Republic of Korea).

### Enzyme-linked immunosorbent assays (ELISA)

Urinary ACTH concentrations were measured using ELISA kits (MyBioSource, Inc., San Diego, CA).

### Western blot analysis

To quantify protein levels, equal amounts of total protein were loaded into lanes and separated on SDS-polyacrylamide gels. The gels were transferred to Immobilon-P membranes (Millipore, Billerica, MA, USA) and the membranes were then blocked with 5% nonfat milk in Tris-buffered saline containing 0.05% Tween 20 (TTBS) at room temperature for 1 h and incubated overnight at 4°C with a primary antibody diluted 1:1000 to 1:5000 in 5% nonfat milk in TTBS. The primary antibodies used were antisera against AMPK and p-AMPK (Cell Signaling, Danvers, MA, USA), LXRα (Abcam, Cambridge, UK), and POMC (Novus Biologicals, Littleton, CO, USA). Horseradish peroxidase-conjugated anti-rabbit or anti-goat antiserum was used as a secondary antibody (1:5000), and antigen-antibody complexes were visualized using an Enhanced Chemiluminescence Plus Kit (GE Healthcare, UK), followed by exposure to X-ray film.

### Immunoprecipitation

Cell lysates were incubated with 5 μl of anti-phospho-Threonine antiserum (Cell Signaling, Danvers, MA, USA), or preimmune serum at 4°C for 16 h. Immune complexes were further incubated with protein A/G-Sepharose beads (GE Healthcare, UK) at 4°C for 2 h. Immunocomplexes were eluted by boiling for 10 min in a sample buffer containing 2% SDS and 10 mM dithiothreitol, separated on SDS-polyacrylamide gels, and then immunoblotted using anti-LXRα antibody.

### Immunohistochemistry (IHC)

Paraffin-embedded pituitary sections (4 μm) were rehydrated and autoclaved at 121°C for 10 minutes in 100 mM citrate buffer (pH 6.0) for retrieving antigens prior to staining. The sections were then treated with 3% hydrogen peroxide for 30 min and with 10% bovine serum for 2 h to block nonspecific binding. They were then incubated with antibodies against p-AMPK (1:20; Cell Signaling Technology, Danvers, MA, USA), POMC (1:100; Novus Biologicals, Littleton, CO, USA), ACTH (1:100; Novus Biologicals, Littleton, CO, USA), LXRα (1:50; Santa Cruz Biotechnology, Santa Cruz, CA, USA), or LXRβ (1:50; Santa Cruz Biotechnology, Santa Cruz, CA, USA) over night at 4°C. Biotinylated secondary antibodies (Vector laboratories, Burlingame, CA, USA) were used for staining p-AMPK (1:50), POMC (1:200), ACTH (1:200), LXRα (1:200), or LXRβ (1:200). The immune complexes were visualized using the Vectastain ABC kit (Vector Laboratories, Burlingame, CA). Negative controls were performed using IgG isotype antibodies (eBioscience, San Diego, CA). To standardize color development, the incubation time for diaminobenzidine staining was fixed in all experiments. All immunostained sections were lightly counterstained with hematoxylin. The slides were evaluated with a bright-field microscope (BX-51; Olympus, Tokyo, Japan) equipped with a camera (DP70; Olympus, Tokyo, Japan) and a micrograph field of view of the entire stained section. Relative intensities of stained targets were calculated using ImageJ v1.47 (NIH, USA).

### Statistical analysis

All data were analyzed using IBM SPSS Statistics 19 (Chicago, IL, USA), and results are expressed as means and standard deviations. Data were statistically analyzed using the Wilcoxon signed ranks test, Pearson correlation, and linear regression. Differences were considered significant when *P* was < 0.05 in two-tailed statistics.

## Author Contributions

K.C., J.-Y.C. and J.Y.C. participated in study concept and design, acquisition of data, and interpretation of results. J.Y.C., S.K.C., I.-J.J. and K.-S.Y. supported clinical study and sample collection. H.-W.S. and J.-W.P. supported mechanism study. K.C. drafted the article, and all authors reviewed and revised the manuscript.

## Supplementary Material

Supplementary Information Antihyperglycemic mechanism of metformin occurs via the AMPK/LXRα/POMC pathway
