# Supplementary material for: Antihyperglycemic mechanism of metformin occurs via the AMPK/LXRα/POMC pathway
Source: Sci Rep. 2015 Jan 30;5:8145. doi: 10.1038/srep08145 (PMC4311245; doi:10.1038/srep08145)
Supplement: Supplementary Information — Antihyperglycemic mechanism of metformin occurs via the AMPK/LXRα/POMC pathway [file srep08145-s1.pdf]

Supplementary Information

for

Antihyperglycemic mechanism of metformin occurs via the AMPK/LXRα/POMC pathway

Kumsun Cho[1,2], Jae Yong Chung[3,*], Sung Kweon Cho[4], Hyun-Woo Shin[5,6], In-Jin Jang[1], Jong-Wan Park[2,5,6], Kyung-Sang Yu[1,2], Joo-Youn Cho[1,6,*]

[1]Department of Clinical Pharmacology and Therapeutics, Seoul National University College of Medicine and Hospital, Seoul, Republic of Korea

[2]Department of Biomedical Science, Seoul National University College of Medicine, Seoul, Republic of Korea

[3]Department of Clinical Pharmacology and Therapeutics, Seoul National University College of Medicine and Bundang Hospital, Seongnam, Republic of Korea

[4]Department of Pharmacology, Yonsei University College of Medicine, Seoul, Republic of Korea

[5]Department of Pharmacology, Ischemic/Hypoxic Disease Institute, Seoul National University College of Medicine, Seoul, Republic of Korea

[6]Cancer Research Institute, Seoul National University College of Medicine, Seoul, Republic of Korea

* Correspondence: Joo-Youn Cho, PhD. Department of Clinical Pharmacology and Therapeutics, Seoul National University College of Medicine and Hospital, 103 Daehak-ro,

Jongno-gu, Seoul 110-799, Republic of Korea. Phone: 82.2.740.8286; Fax: 82.2.742.9252; E-mail: joocho@snu.ac.kr

* Co-correspondence: Jae Yong Chung, MD, PhD. Department of Clinical Pharmacology and Therapeutics, Seoul National University College of Medicine and Bundang Hospital, Seongnam, Republic of Korea. Phone: 82.31.787.3955; Fax: 82.2.742.9252; E-mail: mekka@snu.ac.kr

Supplementary Table S1. Chemically identified metabolites in urine specimens collected from metformin-treated subjects and untreated subjects.

**Ion mode**	**t_R (min)**	**Observed mass (*m/z*)**	**Formula**	**Fold change**	***p*-value**	**Identity**	**Super class**
ESI+	0.52	118.0863	$C_5H_{11}NO_2$	0.64	0.0398	Betaine	Amino acids
	3.0	161.1074	-	3.0	0.0006	*(unidentified)*	-
	7.0	363.2166	$C_{21}H_{30}O_5$	0.73	0.0003	Cortisol	Lipids
	8.5	463.2676	$C_{26}H_{38}O_7$	2.0	0.0391	Retinyl β-glucuronide	Lipids
ESI-	1.3	245.1139	-	1.8	0.0245	*(unidentified)*	-
	2.9	279.0980	-	1.5	0.0329	*(unidentified)*	-
	9.7	583.3124	$C_{30}H_{48}O_{11}$	1.6	0.0083	Cholic acid glucuronide	Lipids

Supplementary Fig. S1

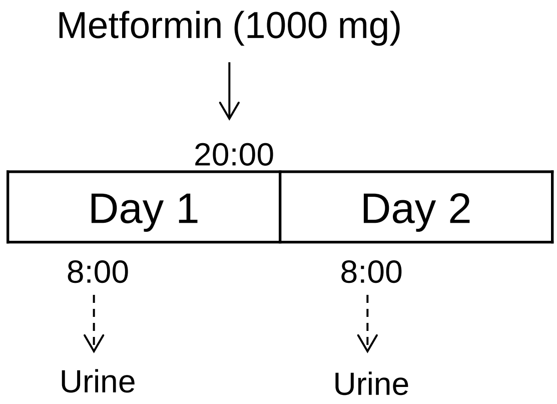

Figure S1. A schedule of metformin administration and urine collection in human study.

Supplementary Fig. S2

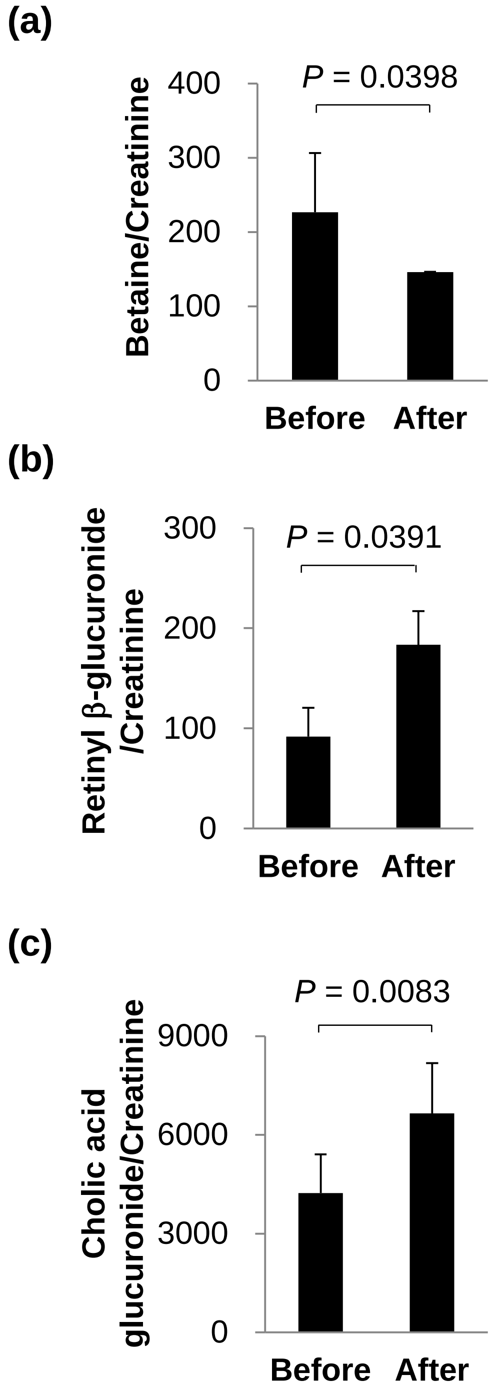

Figure S2. Quantification of identified urinary metabolites in healthy subjects before and after metformin (1000 mg) administration. The normalized urinary concentrations of (a) betaine and (b) retinyl β-glucuronide from ESI+ mode, and of (c) cholic acid glucuronide from ESI- mode. Data are expressed as the mean ± SE. A representative data from 3 independent experiments.

Supplementary Fig. S3

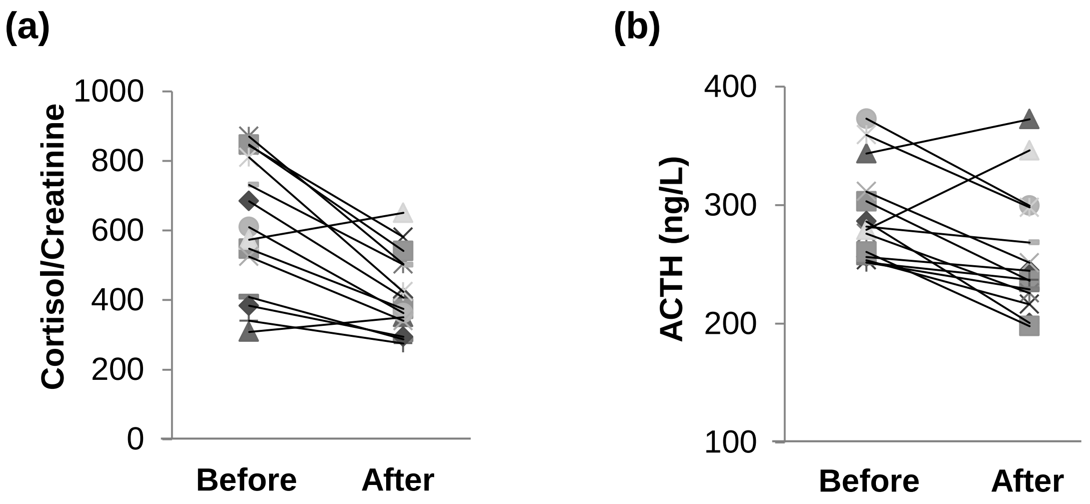

Figure S3. Spaghetti plots for individual subject's urinary (a) cortisol and (b) ACTH levels. A representative data from 3 independent experiments.

Supplementary Fig. S4

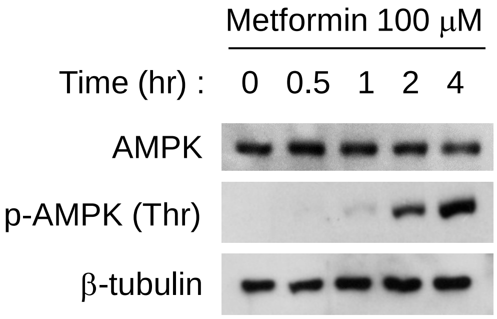

Figure S4. Metformin showed acute phosphorylation of AMPK. The rat pituitary adenoma GH3 cells were treated with metformin by concentration at 10 times higher (100 μM) than we used in main Figures. A representative data from 3 independent experiments. Full-length blots are presented in Supplementary Figure S9.

Supplementary Fig. S5

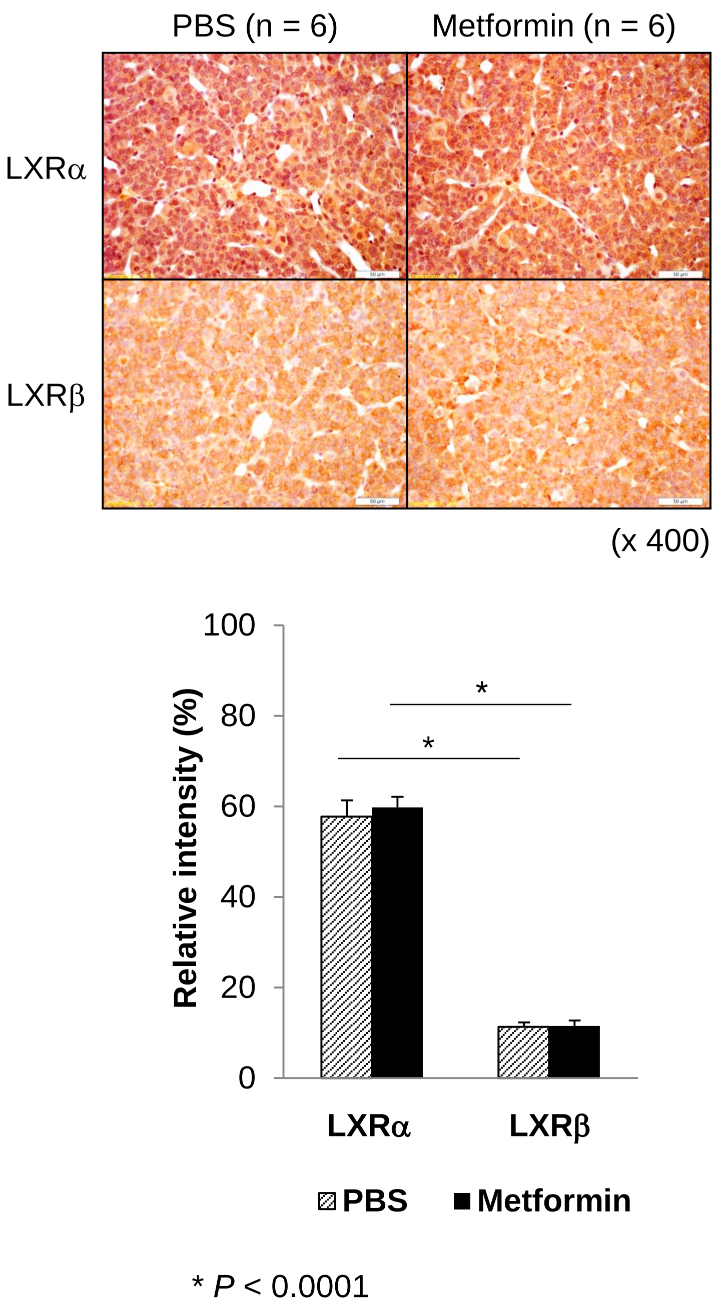

Figure S5. Expression levels of LXRα and LXRβ after metformin treatment *in vivo*. LXRα and LXRβ expression levels in the metformin treatment group were comparable to the control groups. The images are of a representative section (original magnification, ×400. Bar, 50 μm) (*upper*). The number of cells immunoreactive for LXRα and LXRβ was normalized to the total number of cells. Data represent the mean ± SE (n = 6) (*lower*).

Supplementary Fig. S6

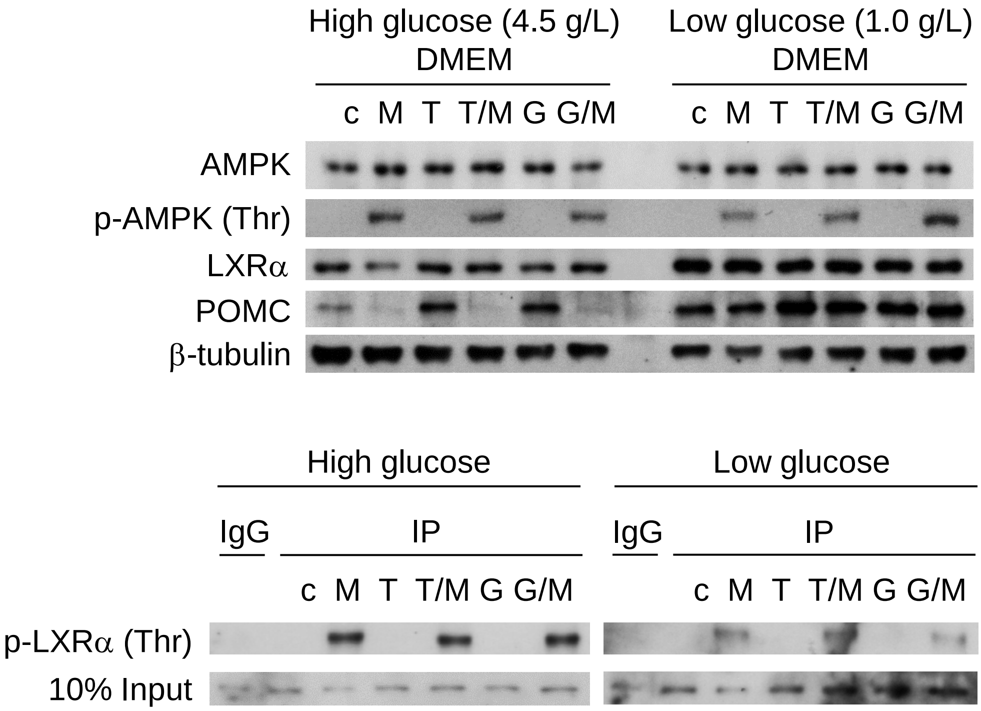

c: control (DMSO)
M: Metformin (10 uM)
T: T0901317 (10 uM)
G: GW3965 hydrochloride (10 uM)

Figure S6. Metformin regulates POMC differently according to the glucose concentration. The rat pituitary adenoma GH3 cells were treated with LXRα agonist either T0901317 (T) or GW3965 hydrochloride (G) or with or without metformin for 8 hours, and total cell lysates were used for western blotting. Metformin upregulated AMPK phosphorylation in both high glucose and low glucose media, however, POMC expressions were different under high or low glucose media condition (*upper).* Total cell lysates were used for immunoprecipitation with anti-phopho-Thr antibody and western blotting with anti-LXRα antibody (*lower*). A representative data from 3 independent experiments. Full-length blots are presented in Supplementary Figure S10.

Supplementary Fig. S7

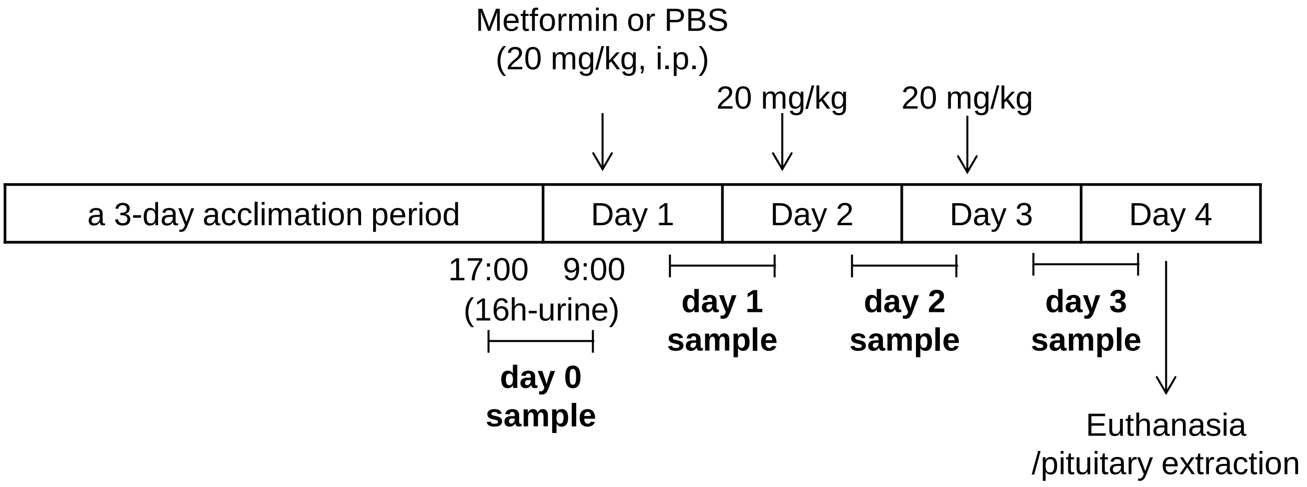

Figure S7. The design of rat experiments: a schedule of metformin administration, urine collection, and pituitary extraction.

Supplementary Fig. S8

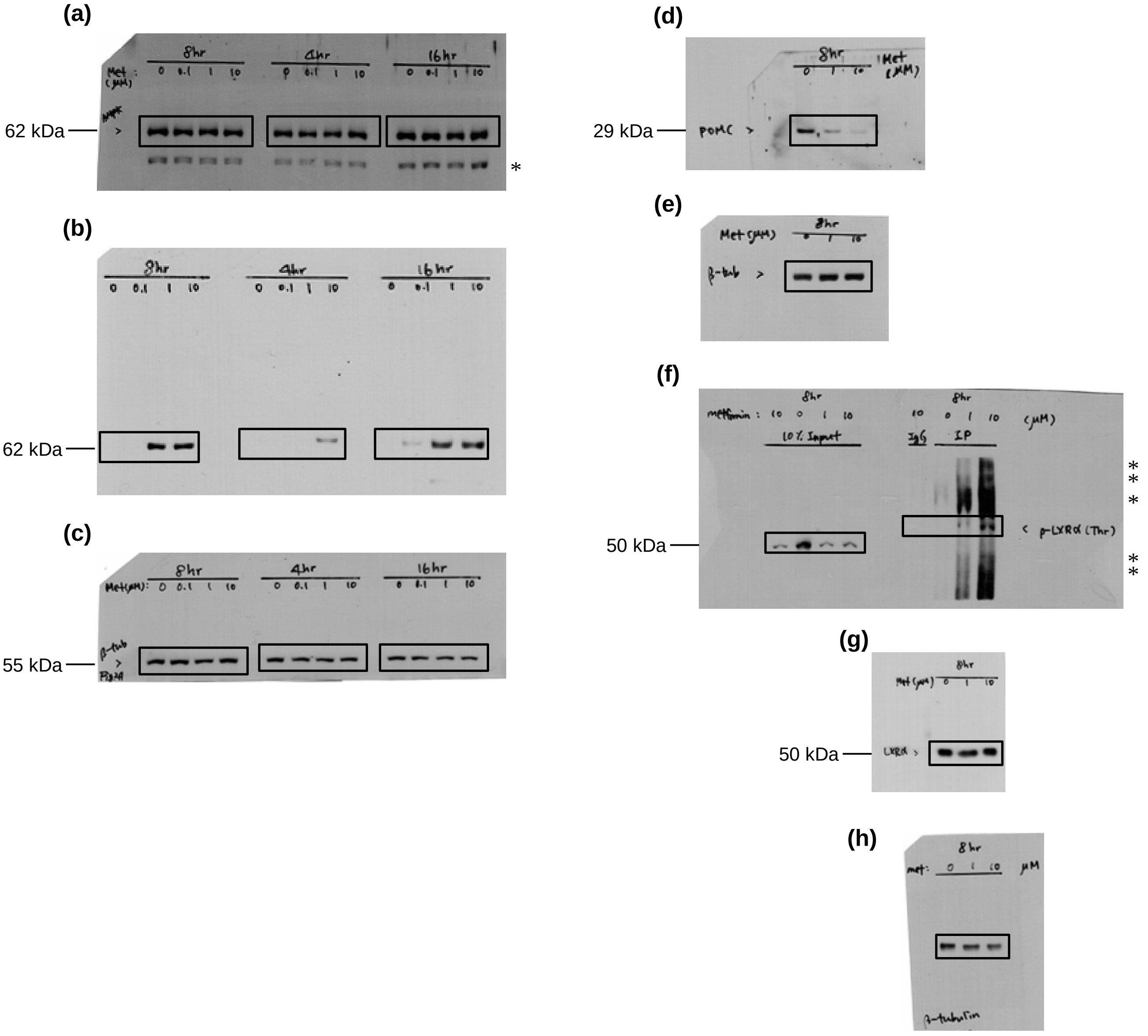

Supplementary Fig. S8

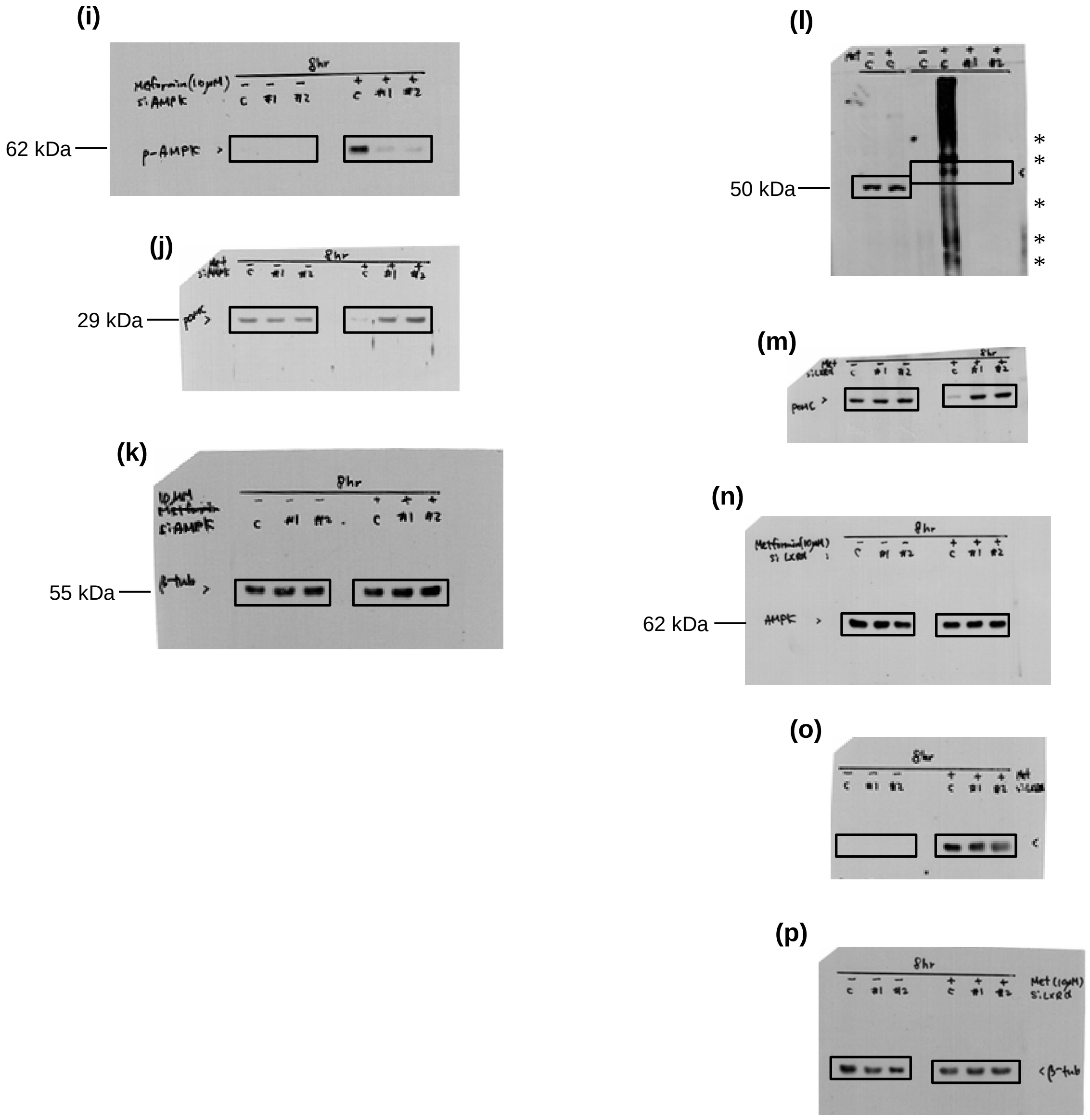

Figure S8. Full-length images of western blots scanned. Rectangular boxes indicate cropped regions used in the representative images. Full-length images of (a) AMPK, (b) p-AMPK (Thr), (c) β-tubulin blots of Figure 3a; (d) POMC, (e) β-tubulin blots of Figure 3b; (f) 10% Input and p-LXRα (Thr), (g) LXRα, and (h) β-tubulin blots of Figure 3c; (i) p-AMPK (Thr), (j) POMC, (k) β-tubulin blots of Figure 3d; (l) 10% Input and p-LXRα (Thr), (m) POMC, (n) AMPK, (o) p-AMPK (Thr), and (p) β-tubulin blots of Figure 3e. A representative data from 3 independent experiments. * Nonspecific bands.

Supplementary Fig. S9

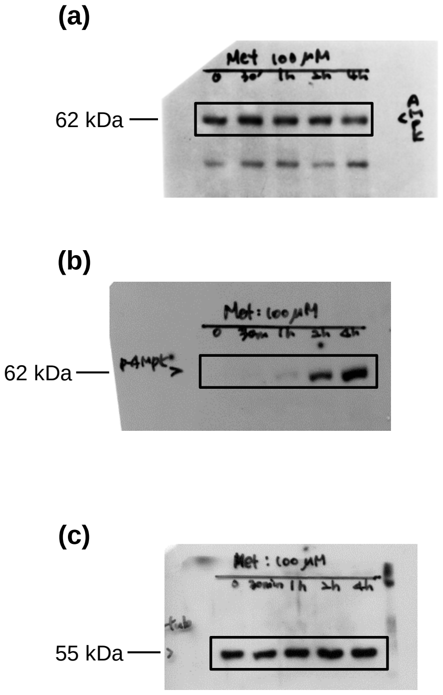

Figure S9. Full-length images of western blots scanned. Rectangular boxes indicate cropped regions used in the representative images. Full-length images of (a) AMPK, (b) p-AMPK (Thr), (c) β-tubulin blots of Supplementary Figure S4. A representative data from 3 independent experiments. * Nonspecific bands.

Supplementary Fig. S10

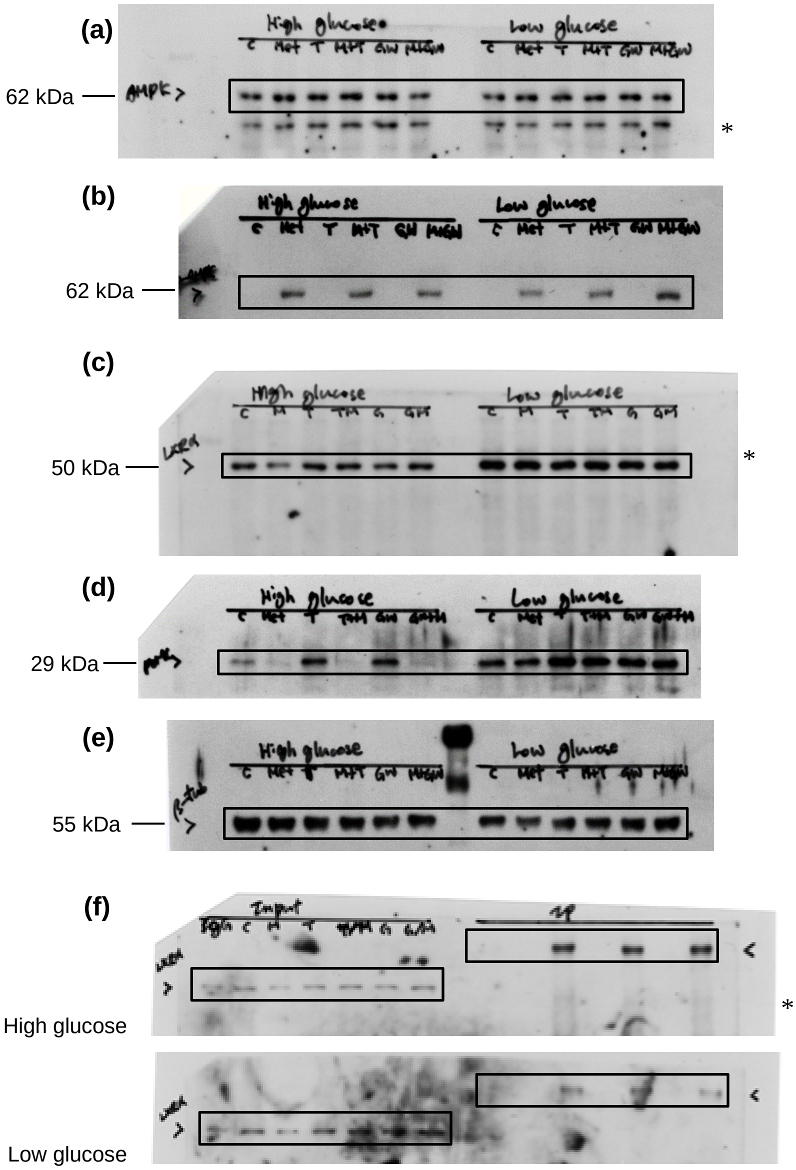

Figure S10. Full-length images of western blots scanned. Rectangular boxes indicate cropped regions used in the representative images. Full-length images of (a) AMPK, (b) p-AMPK (Thr), (c) LXRα, (d) POMC, (e) β-tubulin, and (f) 10% Input and p-LXRα (Thr) blots of Supplementary Figure S6. A representative data from 3 independent experiments. * Nonspecific bands.
